# Performance Evaluation of an Information Technology Intervention Regarding Charging for Inpatient Medical Materials at a Regional Teaching Hospital in Taiwan: Empirical Study

**DOI:** 10.2196/16381

**Published:** 2020-03-25

**Authors:** Min-Chi Liao, I-Chun Lin

**Affiliations:** 1 Department of Industrial Engineering and Management National Yunlin University of Science and Technology Yunlin Taiwan; 2 Department of Nursing National Taiwan University Hospital Yunlin Branch Yunlin Taiwan

**Keywords:** Information System Success Model, information technology intervention, charging, medical materials, work performance

## Abstract

**Background:**

The process of manually recording the consumption of medical materials can be time consuming and prone to omission owing to its detailed and complicated nature. Implementing an information system will better improve work performance.

**Objective:**

The Information System Success Model was adopted as the theoretical foundation. The opinions of nursing staff were collected to verify the impact of the system intervention on their work performance.

**Methods:**

This cross-sectional study was conducted at a regional teaching hospital. Nursing staff were invited to participate in the field survey. A total of 296 questionnaires were collected, and of these, 284 (95.9%) were valid and returned.

**Results:**

The key findings showed that two critical factors (“subjective norm” and “system quality”) had significant positive effects (both *P*<.001) on user satisfaction (*R*^2^=0.709). The path of “service quality” to “user satisfaction” showed marginal significance (*P*=.08) under the 92% CI. Finally, the explanatory power of the model reached 68.9%.

**Conclusions:**

Support from the top management, appointment of a nurse supervisor as the change agent, recruitment of seed members to establish a pioneer team, and promotion of the system through the influence of opinion leaders in small groups were critical success factors needed for implementing the system in the case hospital. The target system was proven to be able to improve work performance, and the time saved could be further used for patient care, thereby increasing the value of nursing work. The positive experiences gained from this study could lay the foundation for the further promotion of the new system, and this is for future studies to replicate. The example of the successful experience of the case hospital could also serve as a reference for other hospitals in developing countries like Taiwan with regard to the promotion of nursing informatization.

## Introduction

### Background

Computers having high performance are utilized in many industries to increase competitiveness and quality. Informatization has become one of the critical factors determining the success of a company. Information technology (IT) has been heavily introduced in many companies, and the highly complex medical industry is no exception. The National Health Insurance (NHI) program implemented in Taiwan in 1995 not only changed the existing medical ecology, but also affected the development of hospital information systems (HISs). Owing to the impact of changes in the medical payment system and the demand for improved medical quality, medical institutions are facing an enormous challenge. According to the provisions of the NHI Administration, medical expenses must be declared electronically. To comply with the health insurance declaration work, medical institutions had to immediately computerize their operations at that time. In the early days of computerization, the major objective of the HISs established by medical institutions was to focus on collecting health insurance declaration data. Owing to the subsequent increased reliance on IT for daily operations and other demands, such as compliance with hospital evaluation and improvement of medical service quality, other relevant functions were gradually added to the HIS [1]. The NHI system now covers 99.6% of Taiwan’s population, and it has service contracts with 93% of the country’s hospitals and clinics [2]. Health insurance declaration is the hospital’s primary income source, although the hospital’s primary revenue comes from diagnostic and treatment services provided by physicians. Therefore, the initial phase of hospital informatization in Taiwan mainly focused on outpatient clinics and physicians, and computerization and informatization for inpatients and nursing care were only gradually implemented afterwards.

IT and network communications are widely used in various industries, and the communications as such have led to different levels of digital divide owing to differences in resource allocation, learning environments, and even urban-rural gaps [3]. Research in this field has shown that digital divide among hospitals mostly arises from different geographical locations and hospital levels [4]. Meanwhile, female gender and age are two of the major factors causing digital divide [5]. The study conducted by Venkatesh et al in 2003 [6] demonstrated that female gender and age are factors that highly influence user acceptance of new information systems (ISs). Nursing staff are mostly female, and they are disadvantaged regarding adaptation to digital technology because of their low exposure. Thus, they are more likely to encounter problem-solving difficulties when working with ISs [7]. The case hospital is located in an agricultural city in central Taiwan. It is a regional teaching hospital with approximately 941 beds and is a typical rural hospital in Taiwan. Compared with other metropolitan hospitals, the adoption and maturity of IT are comparatively low, and the information literacy of the nursing staff is relatively inadequate. The implementation of informatization in the inpatient nursing department of the case hospital started recently, and prior to this, most procedures were manually operated. For example, regarding charging for inpatient medical materials in the past, the consumption items were manually recorded on charging sheets by nurses after performing various treatments for patients, and the sheets were sent to clerks for manual charging and verification. In addition, nursing work showed several characteristics, including 24-hour continuity of care, a three-shift system, and a high patient-to-nurse ratio. In the case hospital, each nurse needs to take care of 8 to 10 patients in a typical day shift, 13 to 17 patients in a typical night shift, and 16 to 20 patients in a typical graveyard shift. Additionally, the nursing staff has a heavy workload, and nursing work is cumbersome. Thus, manual operations are prone to errors. There are often many disturbances in the ward. For example, when there is an emergency involving an inpatient, the nurse must drop the work at hand and immediately provide emergency care. This results in sudden interruption of the nursing work at hand. Furthermore, there are many treatment items to be managed, and the charging work is cumbersome. With this in mind, these factors may easily cause charging mistakes (eg, missed records, incorrect records, etc). Moreover, manual operations lack efficiency, and this negatively impacts hospital income.

Charging refers to the information included in the record of all financial transactions. Hence, the analysis and processing of charges can serve as a reference for decisions made by managers. To be specific, correct charging can not only reduce and prevent the risks of using expired medical materials, but also scrutinize medical shortage. Patient safety can be ensured, and the hospital costs can be reduced [8,9]. Correct charging and its analysis can facilitate hospital management and decision-making processes, as they will enable hospitals to understand the immediate need or demand for medical materials in each department, construct performance indicators [10,11], develop profit models, and plan hospital development policies [12,13]. Charging is based on the concept of structuralization, wherein an intervention IS can hopefully improve the efficiency of procedures and reduce workload. Because of the construct of structuralization and informatization of charging, numerous benefits can be introduced in hospitals. It can also simplify the charging procedure and provide flexibility for the use of the charging information effectively and efficiently. Work efficiency will therefore be increased. The same is the case for timely and accurate transmission of information [14]. Liu et al [15] aimed to improve the problem of missed charging information by improving the accuracy of wound care declaration in the emergency department and lowering the error rate of wound image data archiving. An app into which image data could be uploaded was first introduced in the emergency room. The data were transmitted to the electronic medical record (EMR) system for automatic archiving. This further solved the problem of omission of file uploading by emergency nurses owing to their busy schedule or work interruption. It also helped reduce declaration errors due to inaccurate archiving. Apart from reducing the workload of emergency nurses, this approach increases the accuracy of health insurance declaration for wound care and reduces revenue loss. As nurses are requested to provide patients with frontline care 24 hours a day, they are most familiar with the use and consumption of medical materials. The introduction of IT in nursing work would enable nurses to complete their duties more efficiently [16,17]. Moreover, an IS could allow instantaneous, cross-temporal, and cross-departmental integration of information. This integration would allow effective cross-departmental communication and co-ordination to occur. Thereby, the overall operational performance of the hospital would be enhanced to a great extent [18].

In view of the high importance of accurate charging and the potential deficiencies with manual charging in the past, the case hospital implemented an inpatient charging system to improve efficiency and effectiveness. However, in the literature, there is a lack of reports discussing whether ward nurses can really meet their needs at work with the use of hospital charging systems. Without effective user feedback for the management, user work efficiency may be negatively affected, which, in turn, could affect the performance of the organization. Therefore, understanding the factors impacting both user satisfaction and the IS will be of great research value for improving the effectiveness of IS implementation and management performance [19,20]. The purpose of this research was to adopt the DeLone and McLean 2003 IS Success Model [21]. To that end, “subjective norm” was incorporated as a variable and “work performance” was incorporated as a dependent variable to better look into and understand the factors impacting nurses’ satisfaction with the charging system and their work performance. Meanwhile, it is hoped that this study will help to improve the evaluation of the current status of system implementation in the case hospital, which could serve as a reference for subsequent system optimization. The same is the case for other rural and regional hospitals in Taiwan with backgrounds similar to those of the case hospital and hospitals located in other developing countries with relevant experience in relation to the introduction of the IS provided. More importantly, the results of this study will hopefully make valuable contributions to the completeness of relevant research on nursing informatization.

### Literature Review

#### Prior Efforts of Applying the DeLone and McLean Information System Success Model to Measure Health Care Information Technology Success

The concept of the IS Success Model is based on an IS theory, which is established to provide a comprehensive understanding of IS success by identifying, describing, and explaining the relationships among the most critical six dimensions of success through which ISs are commonly evaluated. The IS success model has been widely cited in thousands of scientific papers, and this model is considered one of the most influential theories in contemporary IS research. Initial development of the theory was proposed by DeLone and McLean in 1992 [22]. From more than 180 relevant articles published on IS performance, they deduced the following six dimensions: system quality, information quality, system use, user satisfaction, individual impact, and organization impact. Although this model provided the key factors for consideration in academia with regard to IS success, some subsequent scholars, such as Seddon and Kiew [23], Seddon [20], and Pitt et al [24] raised some questions and proposals. In response to this, Pitt et al [24] pointed out that the model was too product oriented and that IT departments provide not only products but also services. Seddon [20] believed that the causal relationship of the model could rather confuse researchers. Therefore, DeLone and McLean [21] further refined the model a decade later in response to feedback received from other scholars working in the IS discipline [21,22]. The revised IS Success Model first included “services quality,” and “use” was appropriately modified to “intention to use,” with an explanation that “intention to use” was an attitude, whereas “use” was a behavior. Meanwhile, “use” and “user satisfaction” were posited to be interrelated, that is, “use” would lead to “user satisfaction,” whereas “user satisfaction” would indirectly affect “use” through “intention to use.” In doing so, a causal bidirectional relationship between “use” and “user satisfaction” existed. Finally, to adapt to the e-commerce environment, “net benefit” was used to indicate whether the overall result of using the IS was positive or negative. Collectively, the core of the IS Success Model is to enhance user perception regarding functional aspects, information processing and output, and service quality of the system through three major quality dimensions in order to strengthen the intention to use and satisfaction of the system, thereby promoting work performance (eg, improve work effectiveness, reduce errors, save time, etc). Specifically, “system quality” refers to the completeness of the system itself, and the indicators measured include response time, functional utility, ease of operation, compliance with users’ needs, flexibility, accuracy, reliability, accessibility, and system integration [21,25]. “Information quality” is related with the quality of output information from the system, and the indicators measured include validity of information, preciseness, immediacy, completeness, relevance, and ease of understanding [21,22,25,26]. Service quality is derived from the well-known SERVQUAL scale [27], and the indicators measured include the components of tangibility, reliability, responsiveness, assurance, and empathy. High quality of service provided by the staff or suppliers is also suggestive of the capability and good intention of the service providers [28]. Pitt et al [24] revised the SERVQUAL scale to measure user perception and evaluation regarding the assistance or service provided by IT departments or system suppliers. Scholars deeply believe that IT departments or system suppliers should not only build and maintain ISs, but also provide services, such as problem solving, education, and training resources, to users [21,29]. User satisfaction is defined as the evaluation of a user’s response after using the output information, and it is an important indicator for measuring IS effectiveness. The indicators measured include system interface satisfaction, software satisfaction, information satisfaction, decision-making satisfaction, and overall satisfaction of the system [21,30].

A review on health care information technology (HIT)–related research has shown that scholars mainly use the IS Success Model as a theoretical foundation to understand the impact of HIT on user behavior, and they have achieved good explanatory power [31-34]. Hwang et al [29] explored the factors considered by physicians regarding the use of EMRs from the benefit perspective. Bossen et al [32] adopted the IS Success Model to evaluate the outcomes of implementing an electronic health record (EHR), which involves conducting a survey among physicians and nursing staff. They found that implementing an EHR enabled hospital staff to grasp patients’ conditions in a timely manner. Hsieh and Su [33] extended the IS Success Model and explored the key success factors of EMRs (impacts of implementation of EMRs from the perspective of medical and health information managers by combining an updated version of the DeLone and McLean IS Success Model [21]). Both Huang et al [35] and Chang and Lin [34] modified the IS Success Model to best explore the impact of introducing an IS to the shift system on the performance and satisfaction of nursing staff.

Furthermore, in the IS discipline, “user satisfaction” and “system usage or use” are the most commonly used key factors to measure the effectiveness of system implementation [21,22]. Ives et al [19] suggested that the measurement of “use” can only serve as a surrogate indicator for system success under specific conditions. The authors defined user satisfaction as the relative value of the IS perceived by users, which was the sum of user perceptions of different aspects of the IS, the evaluation responses, and the attitude toward the IS [19]. Seddon and Kiew [23] defined user satisfaction as either the pleasant feeling or unpleasant feeling after using the IS. DeLone and McLean [21] pointed out that the relationship between “use” and “performance” was not significant if the users adopted the system in “compulsory” or “involuntary” situations. Under specific circumstances when users were requested to use the system (a common situation was requests by managers to use or comply with the implementation of the informatization policy), “use” was not a suitable surrogate indicator of IS success. In such cases, “user satisfaction” is more suitable than “use” as a surrogate indicator for measuring system effectiveness.

Previous research in the field of HIT often treated “user satisfaction” as a key factor in the success of IS implementation [33,34,36,37]. In the present situation, the inpatient charging system was the hospital’s policy to promote informatization of wards and nursing care, and ward nurses were forced to use the inpatient charging system. Therefore, the “use” dimension was discarded, whereas the “user satisfaction” dimension was retained as an intermediary variable.

Work performance refers to all behaviors or actions related to organization goals, and the behaviors or actions can be measured according to individual proficiency and the different levels of contribution to the organization goals. It is believed that work performance should cover efficiency, effectiveness, and productivity [38]. Kast and Rosenzweig [39] believed that performance should include the efficiency, effectiveness, and participation satisfaction of the employees within an organization. Huang et al [35] explored the impact of introducing an IS to the shift system on nursing work performance and satisfaction. Work performance was defined as “the contribution of individual effort by nurses to the organization’s missions and the interactions with other members within a certain period of time that met the organization’s expectation; the quantity of work completed; and the value and quality of work contribution.” Their research findings showed that the satisfaction obtained by nursing staff from using the IS shift system improved work performance to some extent. This part of the results is in line with the findings of the study by Chang and Lin [34]. According to the aforementioned literature, this study used “work performance” as a dependent variable to replace the “net benefit” dimension. The aim of this study was to explore the impact of the introduction of the new inpatient charging system on the performance and satisfaction of nursing staff.

#### Social Norm and Prior Health Care Information Technology–Related Research

Fishbein and Ajzen [40] defined “subjective norm” as the concept of how an individual experiences the perceptions of others important to him or her. For instance, whether they think that the individual should perform a particular action. Hence, subjective norms (social influence factors) are considered a direct determinant of behavioral intention. Ajzen [41] mentioned that subjective norms are the perceived expectations from others that influence a user to have a particular behavior. Subjective norms refer to the rules, regulations, or instructions designed by society on which one should act. For any specific behavior, society has set or designed norms on how to perform that behavior. Subjective norms refer to the kinds of norms an individual follows owing to social pressure. Many previous IS studies have confirmed that a stronger perception of subjective norms affects users’ behavior when using a new system. Thus, subjective norms are considered important factors affecting users’ acceptance or rejection of a new system [42-45]. Garcia-Smith and Effken [46] proposed the Clinical IS Success Model by integrating the Technology Acceptance Model with the IS Success Model. Specifically, social influence was also incorporated into the model to investigate the key factors for successful IS implementation through the evaluation of multidimensional factors. Their study has become a reference model for evaluating the success of IS implementation during the introduction of nursing informatization.

The medical industry has a high degree of industry specificity. Medical service is characterized by the irreversibility of life. The nature of innovation is uncertain, and the consequences of adopting innovations are often difficult to predict. For these reasons, medical personnel tend to respond to the introduction of innovations with more conservative strategies, such as observing and learning from others’ experiences and opinions. They tend to reduce uncertainty about innovation through information from peers and the related social system. The study by Kuo et al [47] showed that the pressure caused by subjective norms, together with incentive measures, effectively motivated physicians to comply with the promotion of the EMR system. Further investigations revealed that the support and execution abilities of senior managerial staff were the keys to promote EMRs in hospitals. The utilization of influence from managers and peers facilitated the launch of the new IS and improved work performance. Huang et al [48] also pointed out that the attitudes of public health care workers toward the use of public health ISs were greatly affected by subjective norms (including peers and superiors). With regard to patient care, members of the care team provide patients with continuous, accurate, and complete care services by collaborating with each other. Together, the care team solves the patients’ problems and assists with their recovery. As a result, care services are inherently highly mission-dependent. The education and professional characteristics of nursing staff are related to performing nursing work by following physicians’ orders and respecting norms, such as clinical guidelines. Nursing staff members are more compliant than other medical staff, and they will consider the opinions given by important people around them (eg, supervisors and peers) or important communities and members when performing nursing work. In addition, at the beginning of the introduction of the inpatient charging system in the case hospital, the head nurse at each nursing station assigned nurses with higher information literacy as seeds and set up special mission teams to assist the promotion of informatization of the charging system. Based on the above factors, this study also incorporated social norms into the research framework. Together with three quality dimensions, this study utilized multidimensional factors to discuss the satisfaction of ward nurses with the hospital charging system and its impact on work performance.

In summary, the introduction of a nursing-related IS could increase the work efficiency of nurses and thus promote patient safety [49-51]. This study validated the construct, with the aim of providing a more efficient working environment for nurses along with the additional safety and security needed for patients.

## Methods

### Ethical Approval

This study was approved by the Human Research Ethics Committee, National Taiwan University Hospital Yunlin Branch (Institutional/Independent Review Board number: NTUH1063703472).

### Target System Implementation and Operation

The hospital where the study was conducted mainly co-operated with different departmental teams from the IT and nursing departments to introduce the new inpatient charging system designated to improve the way of charging for the use of medical materials. This innovation was to replace the manual procedure. After the introduction of the new system, the huge difference observed was that the procedures needed to charge for care services were simplified to a greater extent. All that the nurses had to do was to check the items shown on the interface by clicking on them. In doing so, the money charged for the use of medical materials was automatically transmitted to the charging system, and because of this, clerks, instead of nurses, were mainly held accountable for the cumbersome and tedious tasks related to managing accounting details and financial statements.

### Research Model and Hypotheses

**Figure 1:**
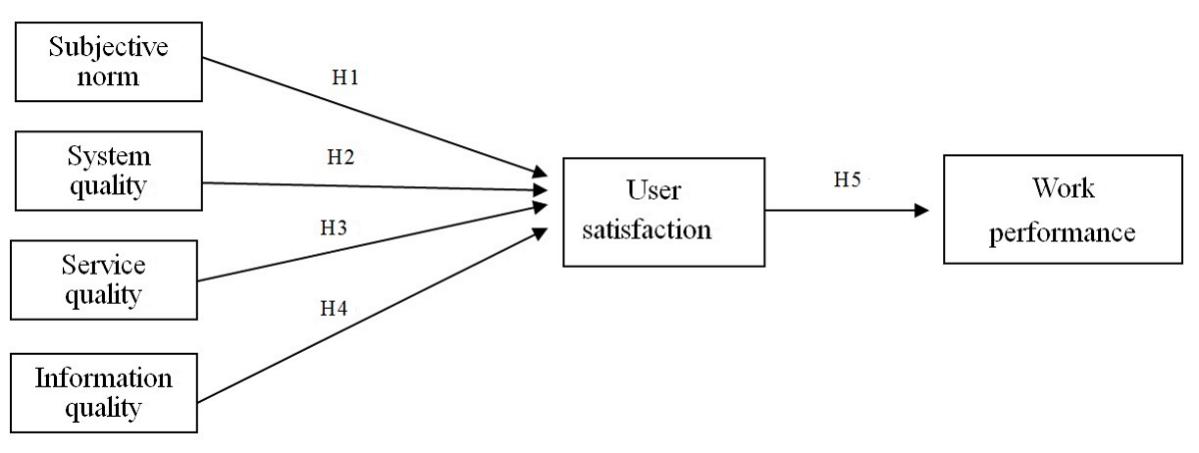
The hypothesized target system success model.

This is a cross-sectional study targeting a regional teaching hospital, which has adopted the e-hospital paperless policy and has implemented the charging system (as the target system mentioned below) for charging medical materials in the inpatient department. Six variables and five hypotheses (Textbox 1) were proposed in our model (Figure 1), including subjective norm, system quality, service quality, information quality, user satisfaction, and work performance. Our hypothetical model was empirically tested using data collected from a field survey. Regarding data collection, convenience sampling was used. Nursing staff who had used the system for at least 6 months or longer were invited to participate in the field survey. Participants who agreed to participate in this study filled out a self-reported questionnaire anonymously to protect their privacy. Thirty-nine items were generated according to our literature review. A 5-point Likert scale from 1 (strongly disagree) to 5 (strongly agree) was adopted in this study. The instrument (Multimedia Appendix 1) was reviewed and approved by two medical and nursing informatics scholars, three management IS scholars, and two nursing department managers. The experts all have more than 10 years of working experience. The items were finalized after modification according to expert suggestions. The scale’s content validity was confirmed by professionals from the academic and industrial fields. A total of 296 questionnaires were administered, and of these, 284 were returned, resulting in a valid questionnaire return rate of 95.9%.

**The research hypotheses of this study.**

H1: Subjective norm predicts nursing staff satisfaction with the target system.

H2: System quality predicts nursing staff satisfaction with the target system.

H3: Service quality predicts nursing staff satisfaction with the target system.

H4: Information quality predicts nursing staff satisfaction with the target system.

H5: The extent to which the work performance of nursing staff improves can be predicted through user satisfaction with the target system.

### Data Analysis

Descriptive analysis and analysis of variance (ANOVA) were performed using IBM SPSS Statistics 20.0 (IBM Corp, Armonk, New York, USA) to enhance our understanding of the sample characteristics. Structural equation modeling was conducted using partial least squares path modeling in the software package Smart PLS version 3.0 (Smart PLS GmbH, Bönningstedt, Germany). Two items with loading lower than the recommended value were iteratively deleted from the model. The path coefficients for the trimmed model were therefore calculated and tested.

The reliability and validity of the measurement model were assessed by its psychometric properties. The psychometric properties of the model were assessed according to internal consistency and convergent and discriminant validity. For reflective indicators, internal consistency was measured according to composite reliability and Cronbach alpha [36], with a recommended acceptable value of 0.70 [37]. Convergent validity was measured according to the average variance extracted (AVE), and it was considered adequate when the AVE of each construct reached 0.50 [39]. Discriminant validity was considered the extent to which a variable is truly distinct from other variables [36]. It was considered acceptable when the square root of the AVE of each construct exceeded the correlation coefficient between the specific construct and others in the model. Discriminant validity was verified by factor loading and cross loading. The loading of an indicator on its assigned variable should be greater than its cross loading on all other variables. Moreover, a structural model is considered to include unobservable latent variables and the theoretical relationships among them [39]. It also suggests how well the theoretical model predicts the hypothesized paths or relationships.

## Results

### Samples

A total of 284 samples were selected, and they consisted of 29 seed members and 255 nonseed members. The analyses were performed as shown below.

### Characteristics of the Participating Nursing Staff

The characteristics of the participating nursing staff are presented in Table 1. We found that the majority of the participants had university degrees (220/284, 77.5%), followed by associate degrees (54/284, 19.0%) and graduate degrees (10/284, 3.5%). In terms of age, the majority of the participants were between 21 and 35 years (241/284, 84.9%), followed by between 36 and 40 years (29/284, 10.2%) and between 41 and 50 years (11/284, 3.9%). With regard to nurse competency advancement, the majority of the participants (113/284, 39.8%) were at the N2 level. In terms of seniority, the majority of the participants (171/284, 60.2%) had less than 6 years of job experience. Among the 284 participants, 29 (10.2%) were serving as seed members, who would be promoting and assisting each unit to launch the target system. Similar to the national data [52] in Taiwan, 95.4% (271/284) of the survey respondents were female. Additionally, 85.6% (243/284) of the nursing staff members were under 36 years of age, 14.1% (40/284) were between 36 and 50 years of age, and only 0.4% (1/284) were over 60 years of age. Overall, our sample characteristics are similar to those of the population of nursing staff members across Taiwan.

**Table 1:** Characteristics of the participating nursing staff (n=284).

Variable		Value, n (%)
**Gender**		
	Male	13 (4.6%)
	Female	271 (95.4%)
**Job title**		
	Nurse practitioner	21 (7.4%)
	Nurse	251 (88.4%)
	Head nurse	12 (4.2%)
**Education**		
	Associate degree	54 (19.0%)
	University degree	220 (77.5%)
	Graduate degree	10 (3.5%)
**Seniority, years of experience**		
	≤2	85 (29.9%)
	3-5	86 (30.3%)
	6-10	57 (20.1%)
	≥11	56 (19.7%)
**Age, years**		
	≤20	2 (0.7%)
	21-25	112 (39.4%)
	26-30	75 (26.4%)
	31-35	54 (19.0%)
	36-40	29 (10.2%)
	41-45	5 (1.8%)
	46-50	6 (2.1%)
	51-60	0 (0.0%)
	≥61	1 (0.4%)
**Nurse competency advancement**		
	N	24 (8.5%)
	N1	82 (28.9%)
	N2	113 (39.8%)
	N3	46 (16.2%)
	N4	19 (6.7%)
**Seed members or not**		
	Seed members	29 (10.2%)
	Nonseed members	255 (89.8%)

### Analysis of Variance

In order to enhance our understanding of the sample characteristics, variance analysis was performed by carrying out one-way ANOVA on “nurse competency advancement” (five levels), “seniority” (four intervals), and “whether users served as seed members for promoting nursing informatization” for the six research variables. The results indicated that there were insignificant differences for “nurse competency advancement” (*P=*.84 for “nurse competency advancement” to “user satisfaction” and *P*=.96 for “nurse competency advancement” to “work performance”) and “seniority” (*P*=.79 for “seniority” to “user satisfaction” and *P*=.84 for “seniority” to “work performance”).

However, regarding “whether users served as seed members for promoting nursing informatization,” significant differences were found for all variables (*P*=.02) (Table 2). Further examination revealed that seed members had higher scores for the averages of the six variables as compared with nonseed members (Table 2). Seed members also evaluated the target system more favorably, showing higher scores in information quality, service quality, system quality, user satisfaction, and working performance.

**Table 2:** Results of analysis of variance and average scores according to whether users served as seed members for promoting nursing informatization.

Variable	*F* value	*P* value	Score, mean (SD)	
			Seed members (n=29)	Nonseed members (n=255)
Subjective norm	9.876	.002	4.3 (0.5)	3.9 (0.6)
System quality	9.263	.003	4.2 (0.6)	3.9 (0.6)
Service quality	12.949	<.001	4.2 (0.6)	3.7 (0.6)
Information quality	12.319	.001	4.3 (0.5)	3.9 (0.6)
User satisfaction	9.418	.002	4.2 (0.5)	3.9 (0.6)
Work performance	10.980	.001	4.1 (0.5)	3.8 (0.6)

### Measurement Model Testing: Reliability and Validity of the Questionnaire

In terms of convergence validity and reliability, the factor-loading values of the dimensions were all greater than 0.6 (Table 3), the AVE values were all observed to be greater than 0.5 (Table 3), and the composite reliability and Cronbach α were all greater than .7, indicating good convergence validity and internal consistency overall. In addition, the Fornell-Larcker criterion of interconstruct correlations and cross-loading were used to confirm discriminant validity (Table 3). The diagonal shown in Table 3 was the square root of the AVE, and its minimum value (0.824) was higher than that of any other correlation coefficient in terms of all the other constructs involved in the test, except for user satisfaction to work performance (0.830). The results of the cross-loading analysis indicated that the scale had acceptable discriminant validity.

According to the ANOVA results, there were significant differences between users who were seed members (n=29) and those who were nonseed members (n=255). The data analyses were performed without the seed members (n=255) and with the seed members (total n=284) separately. This was to further explore whether other related possible outcomes were shown owing to the scale properties, research model, and hypotheses. However, the results indicated high similarity between these two groups. Therefore, we present the analysis results of the nonseed members (n=255).

**Table 3:** Scale properties (n=255).

Variable	AVE^a^	CR^b^	Cronbach α	Interconstruct correlations					
				IQ^c^	PF^d^	SEQ^e^	SN^f^	SYSQ^g^	SAT^h^
IQ	0.834	0.938	.900	0.913					
PF	0.727	0.955	.946	0.676	0.853				
SEQ	0.847	0.971	.964	0.545	0.613	0.920			
SN	0.884	0.958	.935	0.677	0.782	0.494	0.940		
SYSQ	0.678	0.950	.940	0.823	0.745	0.553	0.684	0.824	
SAT	0.957	0.985	.977	0.683	0.83	0.539	0.773	0.765	0.978

^a^AVE: average variance extracted.

^b^CR: composite reliability.

^c^IQ: information quality.

^d^PF: work performance.

^e^SEQ: service quality.

^f^SN: subjective norm.

^g^SYSQ: system quality.

^h^SAT: user satisfaction.

### Path Analysis

The results of the path analysis indicated that three suggested research hypotheses (H1, H2, and H5) reached statistical significance (*P*<.001).

**Figure 2:**
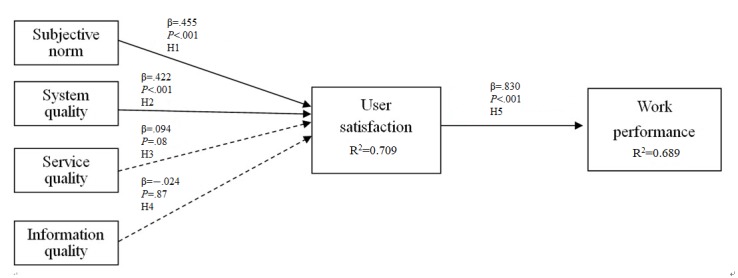
Results of the research model and hypothesis validation.

One path showed marginal significance (*P*=.08) for H3 under the 92% CI. The path from information quality to user satisfaction (H4) did not reach statistical significance (*P*=.87). The path coefficients were 0.455 (*P*<.001) for subjective norm to user satisfaction, 0.422 for system quality to user satisfaction, 0.094 for service quality to user satisfaction, and 0.830 for user satisfaction to work performance (Figure 2). The overall explanatory power of the model reached 68.9%.

## Discussion

### Principal Findings

The demographic information of the participants showed that there was a high proportion of female users (271/284, 95.4%) and that the majority of the participants were between 21 and 40 years of age (270/284, 95.1%). The real-world national data on the nursing population also show that most nurses are between 21 and 40 years of age (114,269/172,897, 66.1%) [52]. The threshold age for marriage is generally estimated to be around 40 years, because this is a suitable age for nurses to start a family. In doing so, they may choose to switch or change their own career or leave the current job owing to the need for child care after marriage or the inability to take turns working in shifts after parental leave. Furthermore, nursing work has the characteristics of work shifts, a three-shift system, and a high patient-to-nurse ratio. Thus, the work is physically challenging for most nursing staff as they grow older. For these aforementioned reasons, the majority of them are between 21 and 40 years of age, and the number of nurses aged over 41 years is gradually decreasing, as age is considered a great challenge if they continue to work as practice nurses.

According to the variance analysis, the nursing staff involved in this study showed relevant differences in their perceptions and evaluations of the target system in terms of all the research variables. Those nurses serving as seed members showed a significantly higher average score as compared with that for nonseed members (Table 2).

We believe that this was related to the fact that nurses who served as seed members were more familiar with the system than those who did not serve as seed members. Seed members were highly involved in the development, discussion, and promotion of the new charging system. With the experience of introducing the new IT charging system, seed members were more likely to facilitate the development of this new system. Their successful experiences could also be considered for replication in further studies regarding the introduction of a new IT system in another medical organization in the future.

The results obtained from the model validation indicated that both the subjective norm and system quality had a significant positive impact on user satisfaction (*R*^2^=0.709, *P*<.001), and thus, these two major factors played decisive roles in the implementation of the target system. Meanwhile, this also demonstrated a positive influence on performance through user satisfaction (*R*^2^=0.689). These results indicate that when a new system is introduced, the positive influence of opinion leaders and the enhancement of users’ awareness and perception of the functional aspect of the system in the initial phase will provide users a chance to perceive the benefits of the new system intervention for their work. This will help increase user satisfaction, which will, in turn, make users more willing to use the system, leading to good work performance. Although the path of service quality to satisfaction exhibited marginal significance (92% CI) and the path coefficient was only 0.094, previous research has already demonstrated the importance of service quality. Therefore, the benefits of improving service quality for the improvement of user satisfaction should not be neglected, as they will, in turn, have positive effects on users’ work performance.

In the case hospital, a chief nursing supervisor was assigned as the change agent when nursing informatization was promoted. In addition to promoting the new system, the change agent also served as a window for communication between the case hospital and the nursing informatization promotion team, as well as the IT department of the headquarter hospital. This dedicated agent also set up a pioneer team by recruiting colleagues with higher information literacy and personalities that were related to being more accepting of changes at each nursing station as seed members. Seed members were the first to receive education and training to familiarize themselves with the system operation. A total of 29 seed members provided successive assistance to promote the new system and remove barriers to system usage for colleagues within the unit. As the case hospital was located in a rural area and more than 95.4% of the nurses were female, the information capability was relatively weak. This was in line with the study conducted by Lin and Lee [7], who pointed out that when most nursing staff are female, there could be a disadvantage with regard to adaptation to digital technology owing to their limited exposure. Thus, it is highly likely that they would face problem-solving challenges when using an IS. Therefore, the case hospital allocated the 29 seed members to various nursing stations to not only guide end users on system operation but also provide immediate assistance to remove barriers when possible. Meanwhile, the case hospital had set up a LINE group chat for communication and interaction. The headquarter IT staff, nurse informaticists, senior management of the case hospital, and all users were invited to join the LINE group chat. All users could voice problems and respond to problems in real time through the LINE group. The attention and support of senior management, appointment of the nurse supervisor as the change agent, establishment of a pioneer team, and effective assistance by seed members who were familiar with the system functions and operations to promote the system enabled users to clearly experience the benefits of the system. These measures effectively improved user satisfaction with the new system, and this outcome corroborated the results from the aforementioned data analysis. For the marginal significance of the path of service quality to user satisfaction, the following reasons are proposed. The new system was mainly developed by the headquarter hospital and was appropriately modified and sequentially introduced in response to the needs of individual hospital units. The IT department in the case hospital had only hardware engineers and no software engineers. All system requirements were collected separately by the chief supervisor, who sent the collected data to the headquarter hospital for modifications. After the system had launched, the chief supervisor was also responsible for collecting opinions and providing feedback to the IT department of the headquarter hospital for further modification. The inability of the IT department to immediately and effectively process users’ opinions and the poor timeliness were the possible reasons why users were unable to form positive perceptions for the immediacy and sufficiency of the assistance and support provided by the IT department. Furthermore, the service quality dimension primarily explored the immediacy, adequacy, and appropriateness of the service, support, and assistance provided by the IT department to the users. This included the completeness of software and hardware resources. However, in the initial phase of system launch, the end users continued to use old computers, which had poor hardware performance. Together with insufficient wireless hotspots, the connection and usage of the system were restricted. We believe that the above reasons may further explain the poor perception of service quality by end users and the marginal significance between service quality and satisfaction.

Furthermore, for information quality and satisfaction not reaching the significance level, we noted and offered the following explanations. Prior to the launch of the new system, the nursing staff performed their tasks at the bedside while ideally manually recording the medical materials consumed. The records were then sent to the clerk, who made each entry individually in the old inpatient charging system for approval. The charging process was highly task-dependent. The principle of the new system design was to provide support for nurses to perform professional work and reduce their workload. The charging work was divided into two parts. The first part mainly involves clicking the consumption items, and the second part mainly involves the automatic system that functions as the mechanism through which the amount of money being paid is shown for subsequent verification and approval. After the new system was launched, the nurses only had to click on the consumed medical materials on the interface to complete the frontend work of charging. Frontline nurses were only able to see the different types of medical materials shown on the interface. On the interface, the amount of medical materials consumed was not shown to the nurses, and thus, they did not need to worry about the calculation of the chargeable amount and the subsequent approval work. Thus, they could not form a perception about the actual calculation of charges. Further processing of the charges and the information output were only managed by clerks. The information quality dimension was mainly used to determine whether the format, immediacy, and accuracy of the processed information presented by the system could satisfy or meet users’ needs or whether the users noticed the changes in these aspects in the new system. In fact, ward clerks were the ones who could most directly perceive the changes in information quality before and after the launch of the new charging system for inpatient medical materials. However, the participants in this study were limited to frontline nurses, and ward clerks were not included. We believe that the above reasons may explain the lack of perception about information quality by nurses, which led to the failure of attaining significance for this path.

A deeper understanding of the factors affecting user satisfaction with the IS will have both research and practical values to improve the effectiveness of IS implementation and management performance. Our study utilized the DeLone and McLean 2003 IS Success Model. “Subjective norm” was chosen as a variable, and “work performance” was chosen as a dependent variable. This was done to understand the factors impacting the ward nurses’ satisfaction with the hospital charging system and their work performance. The model of this study was simple, and the overall explanatory power was 68.9%. This also indicated the feasibility of using the IS Success Model to analyze the effectiveness of a medical-related IS. Additionally, this theory could be widely applied to other areas of studies and so provide a concrete picture of other related research. Furthermore, the current status of system implementation was evaluated through the use of research tools, which allowed the staff in managerial positions to have a better understanding of the main reasons why the lack of significant perceptions about service quality involving the IT department was mainly due to poor hardware performance, insufficient wireless hotspots, and inability to resolve user needs or problems in a timely manner. Therefore, according to the results, the initial steps in the subsequent phase of system promotion are to increase the budget for IT, replace old equipment, and expand wireless hotspots to solve the current urgent problems. Thereafter, users should be provided with better service quality, and the accessibility and availability of the system should be improved. This will increase user satisfaction with the system and thus enhance work support for better work performance. With these factors in mind, the data presented in this study contribute to both practical and research settings.

### Limitations

To explore nursing staff members’ evaluation of the new system and to verify the work performance, this study applied the IS Success Model as a theoretical foundation. However, as with all studies of this magnitude, there were several research limitations, although this case study revealed useful and reliable findings. First, other potential factors were not included. Furthermore, this study was conducted in a case hospital with ward nurses as research participants. The selection of the participants might limit the generalization of the results of this research. Moreover, the case hospital implemented the charging system to replace the existing manual charging procedure performed by nurses. By informatizing the charging system, the charging procedures could be simplified so that nurses are able to record the treatments accurately and perform automatic charging. The conclusions to be drawn are that enhancing system quality, information quality, and service quality could lead to improvements in users’ satisfaction and work performance.

### Recommendations

The introduction of the charging system will require not only user-friendly software but also sufficient IT equipment and a stable wireless connection to be established. In this way, the benefits introduced by using the charging system will be maximized.

### Conclusions

Nursing care is known for its heavy workload. Implementation of the new IT charging system can alleviate nurses’ heavy workload and improve their work efficiency. However, introducing a new system can be a large change to the entire hospital. It may help to break the old habit of using a paper-based approach, which is most familiar to senior hospital staff and administrators. Senior staff and administrators may be strongly suggested to learn how to use various computer-based operations. In other words, implementing the new IT charging system needs the support and encouragement of hospital directors and staff in high managerial positions. The seed members of the new IT charging system will need to pioneer this concept and share their positive experiences about using the new charging system with other hospital units and associates. Thus, smooth and successful promotion of the new IT charging system will be achieved. When the proposed computerized charging system is successfully operated, other medical organizations will be inspired to adopt the same system soon. Thereafter, a nation-wide cloud-based health recording system could possibly be established and operated in the near future. The continuous replication and spread of positive experiences as such encourage and pave the way for the further promotion of the new system in the future. The successful experiences of the case hospital could serve as a reference for other hospitals in developing countries like Taiwan to promote medical and nursing informatization.

## Appendix

Multimedia Appendix 1 Factor loading of each dimension.

## Abbreviations

EHR: electronic health record
EMR: electronic medical record
HIS: hospital information system
IT: information technology
IS: information system
NHI: National Health Insurance

## References

[1] Pang TW (2010). Introduction to Medical Informatics.

[2] Lee PC (2016). 2016-2017 National Health Insurance Annual Report.

[3] Chen WC (2007). A study on information education and digital divide in Taiwan. Journal of Cyber Culture and Information Society.

[4] Glaser J (2007). The electronic health record: a digital divide?. Healthc Financ Manage.

[5] Executive Yuan Investigation report of digital divide in 2008.

[6] Venkatesh V, Morris MG, Davis GB, Davis FD (2003). User Acceptance of Information Technology: Toward a Unified View. MIS Quarterly.

[7] Lin CH, Lee TT (2010). Promoting Nursing Competitiveness: Introduction to the Digital Divide. The Journal of Nursing.

[8] Li MF, Hsiao YS, Lu JW, Lin MH (2014). Reducing Omission Rate For Charging Medical Materials: A Case Study Of A Regional Teaching Hospital. Show Chwan Medical Journal.

[9] Tsai SY, Lei EF, Huang HJ, Chen HL (2015). Improving the Inventory Shortage of Surgical Operational Material through Establishment of an Informational System. Leadership Nursing.

[10] Frandsen A (2010). The role of disciplining/translating accounting practices in patient‐centred care. Intl Jnl Public Sec Management.

[11] Chien CP, Chien TK, Lai WL (2012). Constructing an Internal Control Index System on the Hospital Financial Dimension. Journal of Global Business Operation and Management.

[12] Hou JY, Chang WH, Hsiao CW, Chiang YC (2010). The Application of Power Tracer BIKM to Create a Visual Accounting Chart for Hospital Management-A Case Study of a Regional Hospital in Southern Taiwan. Journal of Healthcare Management.

[13] Maskell BH, Kennedy FA (2007). Why do we need lean accounting and how does it work?. J Corp Acct Fin.

[14] Lim FP (2013). Impact of Information Technology on Accounting Systems. AJMAHS.

[15] Liu CH, Lin IC, Lu JJ, Cai D (2019). A Smartphone App for Improving Clinical Photography in Emergency Departments: Comparative Study. JMIR Mhealth Uhealth.

[16] Chao YL, Lee TT (2015). A Study of the Satisfaction of Nurses with the Clinical Information System in Intensive Care Units. Journal of Nursing and Healthcare Research.

[17] Huang HY, Chou SS, Tseng KJ, Feng RC (2011). Experiences of Bar Code Medication Administration Deployment and Total Quality Management Strategy. Journal of Healthcare Management.

[18] Grabski SV, Leech SA, Schmidt PJ (2011). A Review of ERP Research: A Future Agenda for Accounting Information Systems. Journal of Information Systems.

[19] Ives B, Olson M, Baroudi J (1983). The measurement of user information satisfaction. Commun ACM.

[20] Seddon PB (1997). A Respecification and Extension of the DeLone and McLean Model of IS Success. Information Systems Research.

[21] Delone WH, McLean ER (2003). The Delone and McLean Model of Information Systems Success: A Ten-Year Update. Journal of Management Information Systems.

[22] DeLone WH, McLean E (1992). Information Systems Success: The Quest for the Dependent Variable. Information Systems Research.

[23] Seddon PB, Kiew MY (1996). A Partial Test and Development of Delone and Mclean's Model of IS Success. AJIS.

[24] Pitt LF, Watson RT, Kavan CB (1995). Service Quality: A Measure of Information Systems Effectiveness. MIS Quarterly.

[25] Nelson RR, Todd PA, Wixom BH (2014). Antecedents of Information and System Quality: An Empirical Examination Within the Context of Data Warehousing. Journal of Management Information Systems.

[26] Rainer RK, Watson HJ (2015). The Keys to Executive Information System Success. Journal of Management Information Systems.

[27] Parasuraman A, Zeithaml VA, Berry LL (1988). SERVQUAL: A Multiple-item Scale for Measuring Consumer Perceptions of Service Quality. Journal of Retailing.

[28] Huang MY, Chen TL, Chen YW (2014). Study of Tourism Website Consumers-The Application of Information System Success Model. East-Asia Review.

[29] Hwang HG, Chen RF, Lai YC (2010). Factors Affecting User Satisfaction with Data Warehouse Applications of Taiwanese Banking Industry. Journal of E-Business.

[30] Urbach N, Mueller B (2011). The updated DeLone and McLean Model of information systems success. Information System Theory.

[31] Lau F, Price M, Boyd J, Partridge C, Bell H, Raworth R (2012). Impact of electronic medical record on physician practice in office settings: a systematic review. BMC Med Inform Decis Mak.

[32] Bossen C, Jensen LG, Udsen FW (2013). Evaluation of a comprehensive EHR based on the DeLone and McLean model for IS success: approach, results, and success factors. Int J Med Inform.

[33] Hsieh PJ, Su YH (2015). A Study Exploring Critical Success Factors in the Electronic Medical Record Information System for Medical Information Staff: the Extension of a Successful Model System. Journal of Medical and Health Information Management.

[34] Chang HF, Lin IC (2019). Modeling the impact of nursing shifting system on job performance. The Journal of Taiwan Association for Medical Informatics.

[35] Huang WM, Chen LF, Su YH, Chen CC (2016). The influence on shift-to-shift report information system for the performance and satisfaction. https://mi.oit.edu.tw/m/404-1027-19009.php?Lang=zh-tw.

[36] Petter S, Fruhling A (2011). Evaluating the success of an emergency response medical information system. Int J Med Inform.

[37] Garcia-Smith D, Effken JA (2013). Development and initial evaluation of the Clinical Information Systems Success Model (CISSM). Int J Med Inform.

[38] Campbell JP, McCloy RA, Oppler SH, Sager CE (1993). A theory of performance. Personnel Selection.

[39] Kast F, Rosenzweig J (1985). Organization and Management. A Systems and Contingency Analysis.

[40] Hill RJ, Fishbein M, Ajzen I (1977). Belief, Attitude, Intention and Behavior: An Introduction to Theory and Research. Contemporary Sociology.

[41] Ajzen I (1991). The theory of planned behavior. Organizational Behavior and Human Decision Processes.

[42] Thompson RL, Higgins CA, Howell JM (2015). Influence of Experience on Personal Computer Utilization: Testing a Conceptual Model. Journal of Management Information Systems.

[43] Venkatesh V, Davis FD (2000). A Theoretical Extension of the Technology Acceptance Model: Four Longitudinal Field Studies. Management Science.

[44] Venkatesh V, Morris MG (2000). Why Don't Men Ever Stop to Ask for Directions? Gender, Social Influence, and Their Role in Technology Acceptance and Usage Behavior. MIS Quarterly.

[45] Venkatesh V, Morris MG, Davis GB, Davis FD (2003). User Acceptance of Information Technology: Toward a Unified View. MIS Quarterly.

[46] Garcia-Smith D, Effken JA (2013). Development and initial evaluation of the Clinical Information Systems Success Model (CISSM). Int J Med Inform.

[47] Kuo CY, Zheng SP, Zhang ST, Lai XL, Chan CL (2018). Applying the Conceptual Framework of ?Combined Technology Acceptance Model and Theory of Planned Behavior (C-TAM-TPB) ?to Implement Electronic Medical Records: An Example in a Regional Teaching Hospital. Journal of Medicine and Health.

[48] Huang WM, Wang YH, Hsu HS (2012). An Empirical Study on the Critical Factors in Public Health Information Systems-Taking the Integrated Delivery System with Diabetes Mellitus (IDS-DM) in Central Taiwan for Example. Journal of Innovation and Management.

[49] Chou SS, Chen YJ, Shen YT, Yen HF, Kuo SC (2019). Implementation and Effectiveness of a Bar Code-Based Transfusion Management System for Transfusion Safety in a Tertiary Hospital: Retrospective Quality Improvement Study. JMIR Med Inform.

[50] Sowan AK, Leibas M, Tarriela A, Reed C (2019). Nurses' Perceptions of a Care Plan Information Technology Solution With Hundreds of Clinical Practice Guidelines in Adult Intensive Care Units: Survey Study. JMIR Hum Factors.

[51] van Dulmen S, Driesenaar JA, van Weert JC, van Osch M, Noordman J (2017). PatientVOICE: Development of a Preparatory, Pre-Chemotherapy Online Communication Tool for Older Patients With Cancer. JMIR Res Protoc.

[52] Ministry of Health and Welfare (2019). Statistics of Nursing Staff in Taiwan December 2019.

