# Supplementary material for: Performance Evaluation of an Information Technology Intervention Regarding Charging for Inpatient Medical Materials at a Regional Teaching Hospital in Taiwan: Empirical Study
Source: JMIR Mhealth Uhealth. 2020 Mar 25;8(3):e16381. doi: 10.2196/16381 (PMC7142745; doi:10.2196/16381)
Supplement: Multimedia Appendix 1 [file mhealth_v8i3e16381_app1.docx]

Annexure A

Factor loadings of Each Dimension

| Variables | Items | Loading |
| --- | --- | --- |
| Information Quality | |  |
| IQ1 | I think the information presentation of the charging system on nursing items, physician orders and billing status is detailed. | 0.910 |
| IQ2 | I think the information output of the charging system on nursing items, physician orders and billing status is reliable. | 0.943 |
| IQ3 | I think the information update of the charging system on nursing items, physician orders and billing status is instantaneous. | 0.896 |
| Work Performance | |  |
| PF1 | Using the charging system has helped to improve my performance. | 0.786 |
| PF2 | The charging system has helped me to improve the completeness of charges. | 0.839 |
| PF3 | The charging system has helped me to improve the accuracy of charges. | 0.876 |
| PF4 | The charging system has helped me to address the issue of missed charges. | 0.876 |
| PF5 | The charging system allows me to instantaneously grasp the charging and billing status of patients. | 0.881 |
| PF6 | The charging system has lessened my personal workload. | 0.871 |
| PF7 | The charging system has alleviated my personal work stress. | 0.864 |
| PF8 | I am satisfied with the functional options available on the charging system. | 0.873 |
| User Satisfaction | |  |
| SAT1 | I am satisfied with the interface design of the charging system. | 0.976 |
| SAT2 | I am satisfied with the information presentation of the charging system. | 0.983 |
| SAT3 | Overall, I am satisfied with the charging system. | 0.973 |
| Service Quality | |  |
| SEQ1 | When using the charging system, I can ask questions on the LINE group chat and receive immediate help from team members. | 0.896 |
| SEQ2 | If I encounter problems when operating the inpatient charging system, my issues are taken seriously by the LINE group members. | 0.876 |
| SEQ3 | The LINE group for the charging system gives me full support in charging. | 0.934 |
| SEQ4 | The LINE group for the charging systemcan help me to remove operational barriers immediately. | 0.942 |
| SEQ5 | I believe the LINE group members of the charging system have adequate professionalism. | 0.931 |
| SEQ6 | I believe the professionalism of the LINE group members for the charging system is reliable. | 0.920 |
| System Quality | |  |
| SYSQ1 | The charging system is fast. | 0.839 |
| SYSQ2 | The charging system has a simple interface design. | 0.890 |
| SYSQ3 | I think the charging system has a clear interface design. | 0.885 |
| SYSQ4 | I think the charging system has simple interface operations. | 0.819 |
| SYSQ5 | I think the charging system has appropriately designed functional options. | 0.845 |
| SYSQ6 | I think the charging system has comprehensively designed functional options. | 0.851 |
| SYSQ7 | I think the charging system has good correspondence for nursing items, physician orders and automatic billing. | 0.750 |
| SYSQ8 | I think the charging system has very good functions for automatic billing and billing status. | 0.794 |
| SYSQ9 | I think that the charging system is stable. | 0.844 |
| Subjective Norm | |  |
| SN1 | I use the charging system because my supervisor believes using it is important. | 0.928 |
| SN2 | I use the charging system because my colleagues believe using it is important. | 0.966 |
| SN3 | I use the charging system to carry out charging because it has received the approval of the nursing team. | 0.937 |
